# Can Serum ST2 Levels Be Used as a Marker of Fibrosis in Chronic Hepatitis B Infection?

**DOI:** 10.1097/MD.0000000000001889

**Published:** 2015-10-30

**Authors:** Erkin Oztas, Ufuk Baris Kuzu, Neslihan Inci Zengin, Ismail Hakki Kalkan, Fatih Oguz Onder, Hakan Yildiz, Huseyin Tugrul Celik, Meral Akdogan, Mesut Yalin Kilic, Aydin Seref Koksal, Bulent Odemis, Nuretdin Suna, Ertugrul Kayacetin

**Affiliations:** From the Department of Gastroenterology (EO, UBK, IHK, FS, FOO, HY, MA, MYK, AŞK, SK, BO, NS, EK), Turkiye Yuksek Ihtisas Education and Research Hospital; Department of Pathology (NIZ), Turkiye Yuksek Ihtisas Education and Research Hospital; and Department of Biochemistry (HTÇ), Turgut Ozal University, Ankara, Turkey.

## Abstract

Interleukin 33 (IL-33) is a cytokine belonging to the IL-1 superfamily. Soluble ST2 (sST2) binds to IL-33 and by functioning as trap receptor inhibits signal sending to Th2 via transmembrane ST2. Because Th2-type cytokines play an important role in fibrosis, the aim of this study is to determine whether sST2 can be used as a marker of fibrosis in chronic hepatitis B (CHB) patients or not.

The study included 19 healthy controls, 54 patients with CHB, and 14 patients with cirrhosis because of CHB. The aspartate aminotransferase-to-platelet ratio index (APRI) and fibrosis index based on the 4 factors (FIB-4) scores also calculated, and correlations between liver biopsies, sST2 levels, and these scores were analyzed in CHB and cirrhosis patients.

The sST2 levels in patients with CHB were significantly higher than those in the control group subjects (median: 1133 pg/mL vs 762.5 pg/mL, respectively [*P* = 0.035]). In CHB patients, the METAVIR fibrosis score (stages from 0 to 4) showed a moderate correlation with serum sST2 level (*r* = 0.396, *P* = 0.004) and a weak correlation with FIB-4 score (*r* = 0.359, *P* = 0.008), but no correlation with APRI score (*r* = 0.253, *P* = 0.06). The under the curve value of serum sST2 was 0.68, and its prediction of significant fibrosis (METAVIR score ≥2) in values >674 pg/mL had a sensitivity of 91.7% and specificity of 40% (*P* = 0.009). According to multiple logistic regression analysis, only METAVIR fibrosis stage was found to be an independent predictor of serum sST2 elevation in CHB patients (*P* = 0.04).

The sST2 level can be used for differentiating significant fibrosis from mild fibrosis in CHB patients. However, the efficacy of this marker should be verified by larger studies in the future.

## INTRODUCTION

Chronic hepatitis B (CHB) infection is a significant health problem affecting about 350 million people all over the world. Particularly in developing countries, every year about 1 million patients die of late complications of CHB such as cirrhosis, hepatic failure, and hepatocellular carcinoma (HCC).^1,2^ Patients with marked hepatic inflammation and fibrosis are at greater risk for such complications.^3^ Early diagnosis and appropriate antiviral therapy are crucial in order to prevent development of cirrhosis and other complications, most importantly HCC.^4,5^

Currently, liver biopsy is considered to be the golden standard for evaluating liver fibrosis, but it has limitations and cannot be defined to be perfect. It is contraindicated in patients with serious coagulopathy, infection of the hepatic area, or extrahepatic obstruction. Because it is an invasive procedure, it causes pain in 84% of the patients and serious complications such as hemorrhage, pneumothorax, bile peritonitis, and sepsis. In addition, its reliability is limited by its controversial representation of the liver (sampling ratio, 1:50,000) and different evaluation results obtained by intraobservers and interobservers. Apart from complications, liver biopsy can be defined as the best standard but not golden as it does not have ability to establish definite diagnosis for any patient at any time. The sensitivity and specify of liver biopsy in detecting cirrhosis is not well established, but according to recent knowledge liver biopsy has a possibility to give wrong diagnosis in 20% to 25% of patients which is mostly due to the histopathological pattern of the diseases. Patchy fibrosis or different stages of the disease in different regions of the liver bring these limitations. Another disadvantage of the biopsy is that it is not practical for follow-up.^6,7^ Thus, faster, reliable, and noninvasive methods are required for the diagnosis and follow-up of hepatic fibrosis. Within the last 10 years, noninvasive methods for measuring fibrosis such as the aspartate aminotransferase-to-platelet ratio index (APRI) and the fibrosis index based on the 4 factors (FIB-4) have been developed.^8,9^

Chronic hepatitis causing fibrosis is a clinical entity characterized by persistent inflammatory infiltrations and the role of Th2-mediated immune response in foreground. Cytokines of Th1 origin cause a mild fibrosis during their rapid and intense inflammatory response. The Th2 cytokines such as interleukin 6 (IL-6), IL-4, and IL-13 induce fibrogenesis characterized by hepatic stellate cell proliferation and transforming growth factor β synthesis.^10^ IL-33 is a recently recognized cytokine of the IL-1 family. Immunochemical studies have shown that IL-33 is expressed in the tonsils, Peyer's plaques, lymph nodes, different tissues including liver, and nuclei of the endothelial cells of small and big blood vessels. IL-33 functions as a nuclear factor and cytokine. It has been shown that IL-33 is closely related to chromatin and has a transcriptional inhibitory effect.^11,12^

IL-33 is the pro-Th2 cytokine, and its natural ligand is ST2. ST2 is a glycoprotein in the IL-1 receptor family. When IL-33 binds to ST2, there is an increase in the intracellular mitogen-activated protein kinase and in NF-kB signalling. When IL-33 binds to ST2, it enhances the secretion of Th2-originated fibrogenic cytokines: IL-4, IL-5, and IL-13.^12,13^

There are 3 distinct forms of ST2 gene products: the form bound to membrane and consisting of extracellular, transmembrane, and intracellular components (ST2L); the soluble “secreted” form consisting of only an extracellular component (sST2); and the form with no intracellular component (vST2). ST2L, besides being expressed by Th2 cells, is also expressed by mast cells, macrophages, dendritic cells, natural killer cells, and activated polymorphonuclear leucocytes (PMNLs). On the contrary, sST2 is expressed and secreted by activated PMNLs, respiratory tract epithelium, endothelium, keratinocytes, dermal fibroblasts, and cardiac fibroblasts. When these fibroblasts are activated, they start to express IL-33.^13,14^

It has been reported that sST2 gene expression obviously increases in the pulmonary fibrosis model induced by bleomycin.^15^ In “remodelling” conditions such as myocardial infarction and cardiac insufficiency, the high sST2 levels have been found to be related to mortality and thus claimed to be used as a prognostic marker.^16,17^ It has also been claimed that sST2 levels can be a predictor of the severity and progression of many diseases including inflammatory intestinal disorders.^18^

In comparison to normal liver, the mRNA expression of IL-33 and ST2 significantly increases with the severity of fibrosis, and likewise, a similar increase is seen in cirrhotic liver.^12^ In a mouse model of hepatic fibrosis, the blockage of sST2 by using special antibodies enhances the severity of fibrosis. sST2 has been claimed to be a trap receptor for IL-33.^19^ Experimental studies on mice have reported that sST2 shows a protective effect against injury caused by ischemia/reperfusion by decreasing the proinflammatory cytokine expression in the colon and liver.^20,21^

According to the data in the literature, the presence of fibrosis in any organ system can be reflected by presence of sST2. However, until now there has not been any study on the relationship between the serum levels of sST2 and severity of hepatic fibrosis in CHB patients. The main goal of the present study is to determine whether there is an association between serum sST2 levels and the degree of histopathologically confirmed hepatic fibrosis in CHB patients or not. We also aimed to compare serum sST2 levels with other non-invasive scoring systems, namely with APRI and FIB-4. Furthermore, another purpose of this study is to evaluate the possible independent risk factors that may be associated with the increased levels of sST2.

## PATIENTS AND METHODS

This study was performed in the Gastroenterology Clinic of the Yuksek Ihtisas Education and Research Hospital between January 2010 and March 2012. The study population consisted of patients with CHB (n = 54), patients with cirrhosis because of CHB (n = 14), and healthy control group (n = 19). The study was approved by local ethic committee, and written consent forms were taken from all subjects of the study population before their participation in the study.

### Patient Population

#### CHB Infection Group

Inclusion criteria of the study are as follows: hepatitis B virus (HBV) surface antigen (HBsAg) positivity for >6 months, HBV-DNA level >2000 IU/mL (CobasAmplicor HBV Monitoringequipment, Roche), alanine aminotransferase (ALT) level normal or high, liver biopsy made for chronic HBV infection, and no former therapy. Exclusion criteria of the study are as follows: coinfection with HCV, delta virus, or HIV; chronic liver disease of other etiology (autoimmune hepatitis, metabolic liver disorders, etc.); <10 portal areas determined in the biopsy specimen; daily consumption of >15 g alcohol; presence/history of HCC or other organ system malignancy; history of chronic inflammatory disorder; use of steroids or chronic use of nonsteroid anti-inflammatory drugs (NSAIDs); presence of comorbidity (diabetes mellitus [DM], hypertension [HT], renal failure, coronary artery disease, etc.); and active infection in any organ system.

#### CHB-Induced Cirrhosis Group

CHB infection patients with cirrhosis diagnosed by clinical, laboratory, radiologic, and endoscopic examinations were included in the study. Patients with typical ultrasound findings (irregular hepatic margins, heterogenous and granular echo pattern of hepatic parenchyme, enlarged portal vein and spleen, etc.), elevated bilirubin and decreased albumin levels, prolonged prothrombin time, thromocytopenia and bi- or pancytopenia which are indirect findings of hypersplenism, radiologic evidences of portocaval shunts, and oesophageal varices were evidences for the development of cirrhosis in a patient with diagnosis of CHB. In order to prevent bleeding and complications, these patients did not undergo liver biopsy. Exclusion criteria of the study are as follows: hepatic failure because of other aetiologies (eg, hepatitis D coinfection or hepatitis C infection, autoimmune or metabolic hepatitis); daily consumption of alcohol >15 g; presence/history of HCC or other organ system malignancy; history of chronic inflammatory disorder; use of steroids or chronic use of NSAIDs; presence of comorbidity (DM, HT, renal failure, coronary artery disease, etc.); active infection in any organ system, bleeding esophageal varices, hepatorenal syndrome, spontaneous bacterial peritonitis, or hepatic encephalopathy already presenting with portal HT complications and portal vein thrombosis.

#### Healthy Control Group

This group consisted of Gastroenterology Outpatient Clinic patients with normal complete blood count and normal blood biochemistry, negative viral markers, normal liver in ultrasonographic examination, and functional dyspepsia as final diagnosis. Exclusion criteria of the study in this group are as follows: history of comorbidity (DM, chronic renal failure, etc.), history of chronic inflammatory disease, presence of active infection, use of NSAID or steroids, and history of malignancy.

### Liver Biopsy

The CHB group of patients underwent ultrasonography-controlled percutaneous puncture (using C17-gauge needle) biopsy of the liver. The biopsy specimens were fixed in 10% formalin, embedded in paraffin, and sections were stained with hematoxylin-eosin-saffron and Masson's trichrome stains. A sample length >1 cm and observing >10 portal areas were accepted as sufficient for examination. All liver specimens were examined and evaluated by 2 independent highly experienced pathologists. The staging of fibrosis (F) and inflammatory activity (IA) were made according to the METAVIR scoring system.^22^

#### Measurement of Serum ST2

The serum samples for the assessment of sST2 in the CHB group were taken just before liver biopsy, in the HBV-related cirrhosis group, simultaneously with routine laboratory tests during routine outpatient controls and in the control group, after establishing the final diagnosis of functional dyspepsia. The serum samples were stored at −80°C until the time of use. The sST2 level in each patient was measured twice according to the guidelines of the manufacturer (ST2/IL-1 R4 Quantikine ELISA Kit-R&D System) as described in the following. The serum sample was diluted with buffer solution at a ratio of 1:1, and then incubated on human ST2 antibody-coated plaques for 2.5 hours. Following automated plaque washing, labelled antihuman ST2 antibodies are added to the plaques. After 45 minutes stopping solution was added and measured up. The highest and lowest detection limits of ST2/IL-1 R4 Quantikine ELISA kit were 31.3 and 2000 pg/mL, respectively.

### Laboratory Parameters

The levels of the total bilirubin (<1.2 mg/dL), aspartate aminotransferase (AST) (<33 U/L), ALT (<33 U/L), alkaline phosphatase (33–105 U/L), gamma glutamyltransferase (5–36 U/L), high sensitive C-reactive protein (hs-CRP) (0–0.5 mg/L), platelets (150–440 ×10^3^/uL), and protrombin time (10–12 seconds) were measured.

### Noninvasive Fibrosis Models Used for Comparison

The formulas used to identify APRI and FIB-4 were as follows^23,24^: APRI = [AST (/ULN)/PLT (10^9^/L)] × 100, FIB-4 = age (year) × AST (U/L)]/([PLT (10^9^/L)] × (ALT (U/L)]1/2).

### Comparisons

Patients with chronic HBV infection (n = 54) were categorized as *no/mild fibrosis* for METAVIR fibrosis stage 0 to 1 patients and *significant fibrosis* for METAVIR fibrosis stage ≥2. Fifty-four chronic HBV infection patients and 14 chronic HBV-related cirrhosis patients (total of 68 patients) were together also divided into 2 categories and renamed as following: *noncirrhotic patients* for METAVIR stage 0 to 3 patients and *cirrhotic patients* for METAVIR stage 4 CHB patients plus CHB-related cirrhosis patients. The sST2 levels, APRI, and FIB-4 scores of these groups were calculated and compared with each other, and correlations of sST2, APRI, and FIB-4 scores with METAVIR fibrosis score were analyzed. Also in CHB patients, to identify independent predictors of elevation of serum sST2 levels, a multiple linear regression model was used.

### Statistical Analysis

The statistical analyses were made by using SPSS 18.0 (SPSS Inc, Chicago, IL) software program. The normal distribution compatibility of the variables was tested with visual (histogram and probability graphics) and analytical methods (Kolmogrorov-Smirov or Shapiro-Wilk tests). The descriptive analyses for normally distributed variables were expressed as mean ± standard deviation. For variables which not normally distributed is used as median and values between quartiles (by using frequency tables for ordinal variables). For the comparison of nonparametric variables in > 2 groups, Kruskal-Wallis test and in subgroup analysis Mann-Whitney U test were used. For the identification of the relation between various groups Pearson correlation test was used. The relation between two categorical variables was determined by Fisher's exact test and χ^2^ test. In the analyses, 95% confidence interval was identified, and *P* < 0.05 was accepted as significant. Separate area under receiver operating characteristic (ROC) curves were drawn for severe fibrosis and cirrhosis. The optimum ST2 value and cutoff values of APRI and FIB-4 were calculated according to maximum Youden index (J = sensitivity + specificity – 1). Based on the optimal cut off values determined for sST2, APRI and FIB-4, the sensitivity and specifity percentages were calculated for distinguishing significant fibrosis cases from the ones with no/mild fibrosis and also to discriminate the cirrhotic patients from noncirrhotic patients. Factors with a *P* value of <0.1 following bivariate correlation analyses were included in the multiple logistic regression analysis. A multiple linear regression model was used to identify independent predictors of elevation of serum ST2 level.

## RESULTS

In the 54 patients with CHB infection, the mean level of HBV DNA was 6.1 ± 1.62 log (10) IU/mL, and 15 (27.7%) of these patients were HBeAg-positive. According to the results of liver biopsy examinations, the mean and median values of the Histology Activity Index were found as 1.8 ± 0.8 and 2.0 (0–3). Of the patients, 2 had F0, 28 had F1, 13 had F2, 8 had F3, and 3 had stage 4 fibrosis. Of the patients, 55.6% (n = 30) had no/mild fibrosis and 44.4% (n = 24) had significant fibrosis. All of the 14 patients with CHB-related liver cirrhosis were under oral antiviral therapy, and only 2 of these patients showed detectable levels of HBV DNA (353 and 75 IU/mL). According to the Child-Pugh score, 8 patients were in class A, 5 patients were in class B, and 1 patient was in class C. The mean and median values of the Model for End-Stage Liver Disease score were calculated as 12.85 ± 6.87 and 11 (7–35), respectively.

The characteristics of the study population are shown in Table 1. Demographic features of the groups were similar to each other. Among all laboratory parameters, only in terms of ALT, albumin and PLT values were statistically significant differences between the 3 groups.

**TABLE 1:**
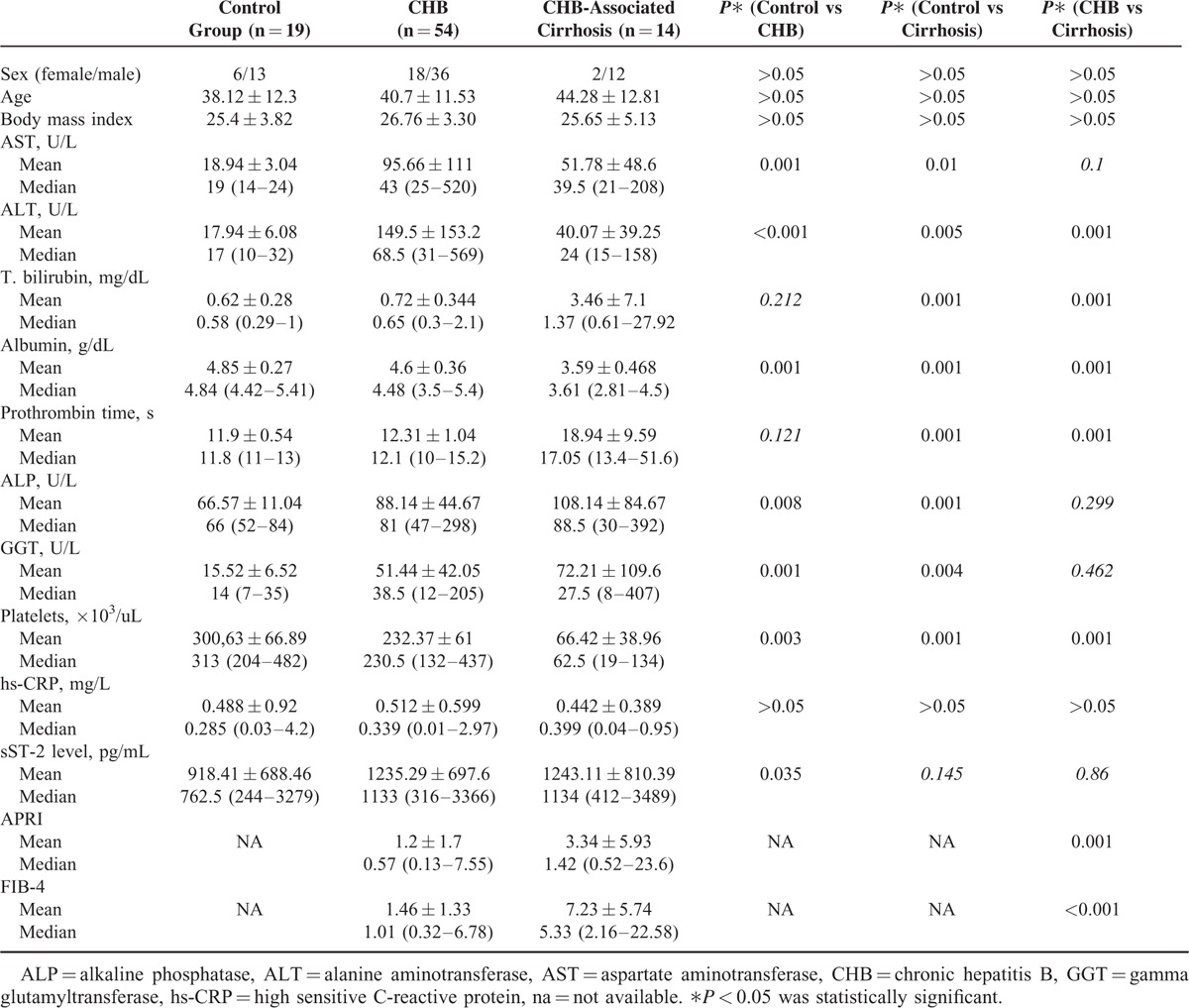
Clinical and Laboratory Findings of the Groups

The mean serum sST2 levels of control group, CHB group, and CHB-related liver cirrhosis group were 918.41, 1235.29, and 1243.11, respectively. The difference in sST2 serum levels between the control group and CHB group was statistically significant (*P* = 0.035); however, the difference between the control group and CHB-related liver cirrhosis group as well as between the CHB group and CHB-related liver cirrhosis group was not statistically significant (*P* = 0.145 and *P* = 0.86, respectively). When CHB and CHB-associated liver cirrhosis patients were taken as a single group (n = 68), the mean serum sST2 levels of this group were significantly higher than control group (1133.25 pg/mL vs 918.41 pg/mL; *P* = 0.033).

**FIGURE 1:**
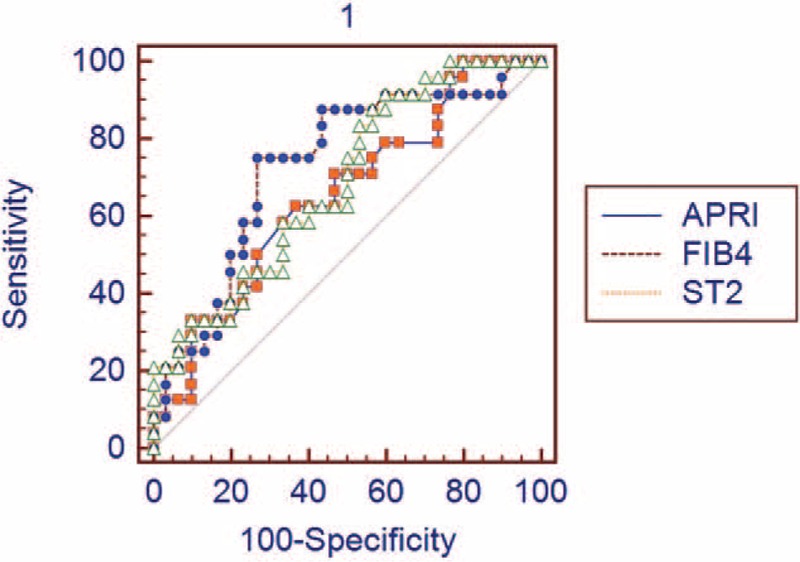
ROC curve analysis of sST2 level, APRI, and FIB-4 for the diagnosis of significant fibrosis. AUC for sST2: 0.68 (95% CI: 0.573–0.827), AUC for APRI: 0.65 (95% CI: 0.511–0.777), and AUC for FIB-4: 0.73 (95% CI: 0.593–0.843). When AUC values were compared, there was no significant difference between the models (serum sST2 vs APRI: *P* = 0.7, sST2 vs FIB-4: *P* = 0.5, APRI vs FIB-4: *P* = 0.1). APRI = aspartate aminotransferase-to-platelet ratio index, AUC = area under the curve, CI = confidence interval, FIB-4 = fibrosis index based on the 4 factors, ROC = receiver operating characteristics, ST2 = Soluble ST2.

Between the CHB group and CHB-related liver cirrhosis group in terms of APRI and FIB-4, scores were different (*P* = 0.001 and *P* =  < 0.001, respectively). In CHB patients, the METAVIR fibrosis score (stages from 0 to 4) showed a moderate correlation with serum sST2 level (*r* = 0.396, *P* = 0.004) and a weak correlation with FIB-4 score (*r* = 0.359, *P* = 0.008), but no correlation with APRI score (*r* = 0.253, *P* = 0.06). On the contrary, the inflammatory activity score showed a weak correlation with ST2 level (*r* = 0.375, *P* = 0.005), a moderate correlation with APRI score (*r* = 0.499, *P* = 0.001) and with FIB-4 score (*r* = 0.552, *P* = 0.001). In the CHB group, when each of the 3 models was analyzed with ROC curve for the differentiation of no/mild fibrosis (F0–F1) from significant fibrosis (F ≥ 2), all models were found to be significant. When the cutoff value of serum sST2 was taken as 674 pg/mL for diagnosis of significant fibrosis (F ≥ 2), its sensitivity and specificity were calculated as 91.7% and 40% (*P* = 0.009), respectively. The cutoff value, sensitivity, and specificity of FIB-4 and APRI score in the diagnosis of significant fibrosis (F ≥ 2) were 1.02%, 75.0%, and 73.3% (*P* = 0.001), and 0.58%, 62.5%, and 63.3% (*P* = 0.04), respectively (Table 2). There was no statistically significant difference between the 3 models in terms of area under the curve (AUC) (Figure 1).

**TABLE 2:**
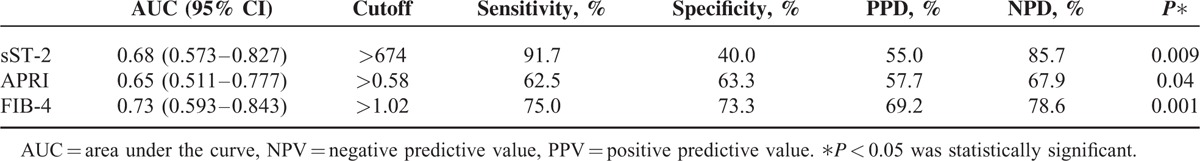
The Diagnostic Performance of Serum sST2 Level and APRI and FIB-4 Scores in the Differentiation of Significant Fibrosis

In CHB patients, the correlation analysis was also made between serum sST2 levels and several probable serum markers indicating severity of liver inflammation: ALT, HBV DNA, hs-CRP, serum total bilirubin, albumin levels, PLT, prothrombin time, and age. Among these parameters, hs-CRP, total bilirubin, prothrombin time, and age showed a significant correlation with serum sST2 levels (*r* = 0.345/*P* = 0.003, *r* = 0.354/*P* = 0.001, *r* = 0.333/*P* = 0.002, *r* = 0.236/*P* = 0.028, respectively). When a multiple logistic regression analysis was performed according to the results of a bivariate correlation test, only METAVIR fibrosis stage was found to be an independent predictor of serum sST2 elevation (Table 3)

**TABLE 3:**
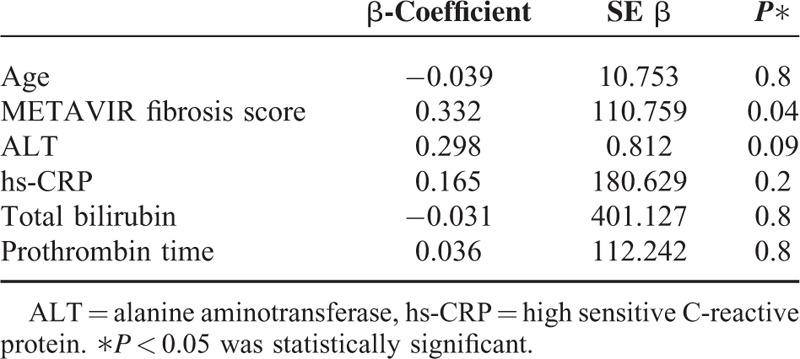
Multiple Linear Regression Analysis to Predict Independent Risk Factor for Elevation of Serum sST2 Level

**FIGURE 2:**
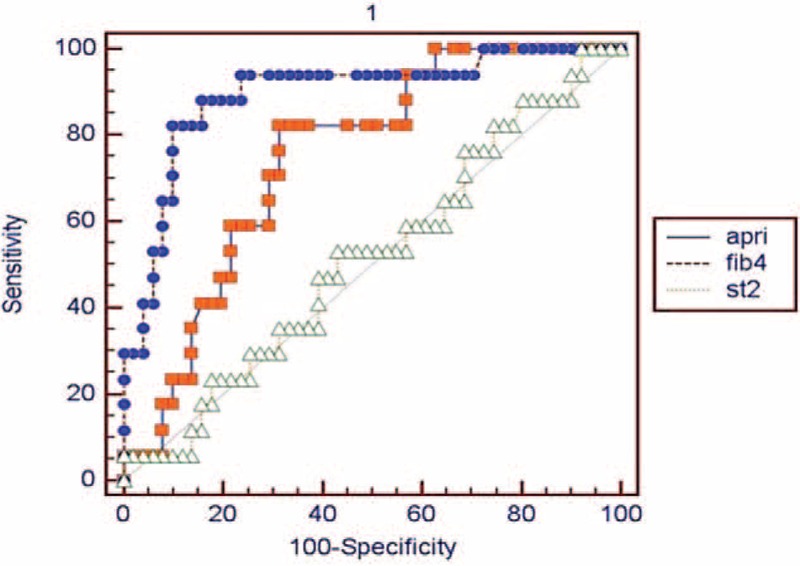
ROC curve analysis of sST2 level, APRI, and FIB-4 for the diagnosis of cirrhosis. AUC for sST2: 0.51 (95% CI: 0.393–0.64), AUC for APRI: 0.74 95% CI: 0.628–0.845), and AUC for FIB-4: 0.89 (95% CI: 0.799–0.957). When the AUC values of the models were compared, FIB-4 score showed a marked superiority to others (serum sST2 vs APRI: *P* = 0.0177, sST2 vs FIB-4: *P*< 0.0001, and APRI vs FIB-4: *P* = 0.0006). APRI = aspartate aminotransferase-to-platelet ratio index, AUC = area under the curve, CI = confidence interval, FIB-4 = fibrosis index based on the 4 factors, ROC = receiver operating characteristics, ST2 = Soluble ST2.

Three patients with METAVIR fibrosis score 4 in the CHB group and 14 patients with cirrhosis were defined as the cirrhotic group (n = 17), and patients with METAVIR fibrosis score <4 were defined as the noncirrhotic group (n = 51). When each of the 3 models was analyzed with ROC curve for differentiation between cirrhotic and noncirrhotic patient groups, only serum sST2 level was found insignificant (*P* = 0.834). For diagnosis cirrhosis, the cutoff value, sensitivity, and specificity of APRI and FIB-4 scores were calculated to be 0.86%, 82.35%, and 68.63% (*P* = 0.0001) and 2.06%, 88.24%, and 84.31% (*P* < 0.0001), respectively (Table 4). Among the determined cutoff values, the AUC of FIB-4 score was more significant than that of APRI score (*P* = 0.0006) (Figure 2).

**TABLE 4:**
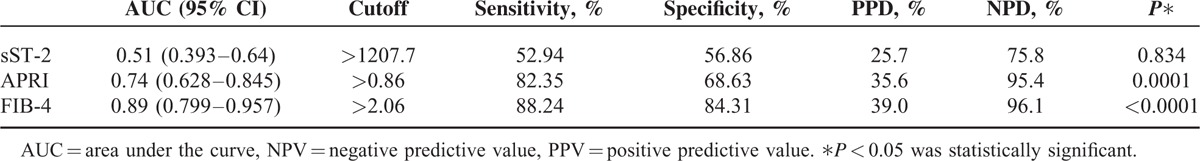
The Diagnostic Performance of Serum sST2 Level and APRI and FIB-4 Scores in the Differentiation of Cirrhosis

## DISCUSSION

Although there are many studies on noninvasive fibrosis models in chronic liver diseases, most of these studies have been made on patients with chronic hepatitis C (CHC) infection. The role of these models in predicting the fibrosis stage has not been fully clarified.^23–25^ The noninvasive fibrosis models have been compared with liver biopsy results, and in contrast to the results in CHC patients, controversial results have been obtained in CHB patients.^26,27^

The inconsistency of noninvasive methods in predicting fibrosis in CHB and CHC patients when compared with biopsy results can be explained by the histopathologic characteristics and course of the infection as described in the following: the regenerative nodules in CHB infection are larger than those in CHC infection; piecemeal necrosis in CHC patients is more localized and less severe than that in CHB patients; and although the progression of fibrosis in CHC is quiet, in CHB it is exacerbated because of acute attacks.^28^ In this context, it can be concluded that even liver biopsy is not a perfect gold standard for determining fibrosis in CHB. Fibrosis screening methods such as fibroscan and magnetic resonance elastography (MRE) might be more helpful in CHB.^29^ However, both ultrasound and MR-based elastography procedures are highly expensive when compared with serum biomarkers and scoring systems. Also, fibroscan is highly affected by determinants caused by both the patient (obesity, nonechogenous patient, and low compliance) and the observer. But serum biomarkers have the same quantitative results far from these determinants.

For comparison with sST2, we choose the APRI and FIB-4 scores. The reason of this choice was that these 2 scores are composed of the routine laboratory parameters, and thus can be readily assessed. We did not include other measurement methods used as clinical and laboratory parameters because we thought that they would not contribute more to the study.

The sST2 levels in CHB patients without cirrhosis were found higher than those in the control group subjects. The sST2 levels in CHB patients with cirrhosis were markedly higher than those in the control group, but the difference was statistically insignificant. This finding was assumed to be due to the limited number of patients in these groups. When cirrhotic patients and CHB patients were evaluated together upon this assumption, the sST2 levels were found significantly higher than those in the control group. Likewise, studies have shown that sST2 levels in patients with acute or chronic liver diseases are higher than those in the controls.^30,31^ When the relation of sST2 levels with liver fibrosis was assessed and compared with APRI and FIB-4 scores, it was found that the sST2 levels had a stronger correlation with METAVIR fibrosis stages than with APRI and FIB-4 scores. Former studies have also reported that APRI score shows no correlation with fibrosis in CHB patients, but shows a strong correlation in CHC patients.^32,33^

The AUC value of the APRI score for the diagnosis of significant fibrosis in CHB patients was found to range from 0.62 to 0.77, and sensitivity and specificity to range from 36% to 89% and from 41% to 80%, respectively.^34–39^ Although there is not a standard cutoff value for the HBV population yet, in these studies generally the cutoff value of 0.5 to 1.5 for APRI score has been used.^34–39^ In these studies, the AUC value for FIB-4 score has ranged from 0.75 to 0.79, and sensitivity and specificity from 25% to 73.5% and from 68.1% to 96%, respectively. For FIB-4, different cutoff values ranging from 1.1 to 3.25 have been used.^34,36,40^ In our study, the AUC values of APRI and FIB-4 in differentiating significant fibrosis were 0.65 and 0.73, with sensitivities and specificities compatible with those in the literature.

The AUC value (0.68) of the serum sST2 level was similar with those of the 2 models, and showed a higher sensitivity and lower specificity for diagnosing significant fibrosis (91.7% and 40%, respectively). This sensitivity value is fairly impressive. Based on these results it is possible to suggest that in every 9 out of 10 CHB patients with serum sST2 levels >674 pg/mL significant fibrosis may be present. However, the specifity for diagnosing significant fibrosis was rather low. Anyway, determining the patients without significant fibrosis, namely the relatively healthy ones, out of the population with CHB, was not one of the objectives of our study. Although the number of the patients involved was relatively low and also it is early to suggest a final decision, these findings seem to fulfill the main aim of the present study. The another striking finding of this study was that; although various parameters together with METAVIR fibrosis stage were evaluated in multiple lineer regression analysis, only METAVIR fibrosis stage was found as an independent risk factor for the increased serum sST2 levels. In other words, serum sST2 levels increase with only increasing METAVIR fibrosis stage. Fibrosis stage is the only predictive factor for elevated serum sST2 levels in CHB patients and vice versa. To the best of our knowledge, no any other single serum markers were reported to date as sST2, which shows a direct relation with fibrosis stage.

However, the AUC value of sST2 concentration (in F ≥ 4 cirrhotic patient group) was 0.512 which was not a statistically applicable value for differentiating significant cirrhosis. On the contrary, the APRI score and particularly the FIB-4 score were found more efficient in the differentiation of cirrhosis, with AUC, sensitivity, and specificity values of 0.74, 82.3%, 68.6%, and 0.89, 88.2%, 88.3%, respectively. Previously reported AUC, sensitivity and specifity values for the differentiation of cirrhosis in CHB patients ranged between 0.64-0.83, 62%-82.3% and 62.5%-74% respectively for APRI score, and ranged between 0.66-0.87, 67%-87.6% and 43.8%-80% respectively for FIB-4 score.^32,34,36,39,41–43^ An important finding in our study was that FIB-4 score was more efficient in predicting fibrosis than APRI score, as reported by other studies.^8,43,44^

The clinical implication of any noninvasive measurement method for differentiating noncirrhotic population from the cirrhotic one is open to discussion. Also, there are many easily accessible methods for cirrhosis diagnosis such as physical examination, routine laboratory tests, ultrasound imaging, and upper gastrointestinal system endoscopy. On account of these methods, differentiation of cirrhosis by any noninvasive method should not be seen as an advantage. As mentioned above, the basic purpose of our study was to measure the capacity of noninvasive tests such as sST2 and APRI/FIB-4 for differentiating significant fibrosis. Significant fibrosis (F2 and over) has been accepted as the most important criteria to begin antiviral therapy both in international guidelines and legally in our country.^45,46^ In this context, sST2 seems to be superior to APRI and FIB-4 in differentiating significant fibrosis. It cannot determine the cirrhotic population, but we think that this is clinically unnecessary.

Limitation of our study can be small number of patients and not using noninvasive imaging methods such as fibroscan and MRE. Liver biopsy and serum sST2 controls after a certain time under antiviral therapy could have contributed more to our study. However, liver biopsy particularly in the follow-up of liver fibrosis is not a practical method that can be repeated many times, and the majority of our CHB patients refused liver biopsy in their follow-up controls. Also, all patients in the study did not have any diagnosis of congestive heart failure according to their medical history and none of them showed any clinical symptom of this disease. However, they were not proven by use of any other specific serum markers such as mid-regional pro-atrial natriuretic peptide, mid-regional pro-adrenomedullin, highly sensitive troponins, and Galectin-3. This may also be a limitation of our study.

In conclusion, our study is the first study to investigate the relationship between serum sST2 levels and fibrosis stages in CHB patients. Our results showed that sST2 had a stronger correlation with METAVIR fibrosis staging than with APRI and FIB-4 scores. In the differentiation of significant fibrosis in cases of F2 stage, sST2 was found to be higher sensitivity that can be used with more success than APRI and FIB-4, but it is seen that in the differentiation of cirrhosis has a low diagnostic performance. By determining that the only independent risk factor for elevated sST2 levels was fibrosis stage in this pilot study, it would be proper to suggest that serum sST2 may be a reliable and simple parameter both for follow-up and in evaluating the response to any treatment modality, not only for CHB but also for all liver diseases causing fibrosis (ie, nonalcolic fatty liver disease). Further studies evaluating the sST2 level as a predictive and prognostic factor in liver diseases and assessing different clinical and laboratory parameters are required.
