# Can Serum ST2 Levels Be Used as a Marker of Fibrosis in Chronic Hepatitis B Infection?: Erratum

**DOI:** 10.1097/01.md.0000476096.25553.94

**Published:** 2015-12-18

In the article “Can Serum ST2 Levels Be Used as a Marker of Fibrosis in Chronic Hepatitis B Infection?”,^1^ which appeared in Volume 94, Issue 47 of *Medicine*, Dr. Fatih Saygili's name was omitted from the byline. The article has since been corrected online.
